# Amelioration of Inflammatory Cytokines Mix Stimulation: A Pretreatment of CD137 Signaling Study on VSMC

**DOI:** 10.1155/2017/1382805

**Published:** 2017-02-09

**Authors:** Wei Zhong, Bo Li, Xiao Yang Li, Zhong Qun Wang, Chen Shao, Cui Ping Wang, Rui Chen, Jin Chuan Yan

**Affiliations:** Department of Cardiology, Affiliated Hospital of Jiangsu University, Zhenjiang, Jiangsu Province, China

## Abstract

Previous studies showed little CD137 expressed in normal vascular smooth muscle cells (VSMCs) and it is important to find a valid way to elevate it before studying its function. The level of CD137 was detected by RT-PCR, western blot, and flow cytometry, respectively. CD137 signaling activation was activated by agonist antibody and measured through phenotype transformation indicators and cell functions. Proteins in supernatants were detected by ELISA. The total CD137 elevates under different concentrations of CM treatment. Among these, 25 ng/ml CM treatment increases the CD137 expression mostly. However, flow cytometry demonstrates that 10 ng/ml CM elevates surface CD137 more significantly than other concentrations and reaches the peak at 36 h. At 10 ng/ml, but not 25 ng/ml CM pretreatment, the levels of phenotype related proteins such as SM-MHC, *α*-SMA, and calponin decrease while vimentin and NFATc1 increase, suggesting that VSMCs undergo phenotype transformation. Transwell, CCK-8 assay, and ELISA showed that the ability of VSMCs viability, migration, and IL-2 and IL-6 secretion induced by CD137 signaling was significantly enhanced by the pretreatment of 10 ng/ml CM. This research suggested that 10 ng/ml CM pretreatment is more reasonable than other concentrations when exploring CD137 function in VSMCs.

## 1. Introduction

CD137, also known as 4-1BB or TNFRSF9, belongs to the tumor necrosis factor (TNF) superfamily (TNFRSF) and is increasingly recognized as a key factor in immune and inflammation responses [1]. CD137 expression is mainly found in T cells, DCs, NKs, and monocytes. Activation of CD137 signal contributes to cytokine production, expansion, and functional maturation which can enhance immune response [2, 3]. Recent study showed that the combined activation of CD137 and programmed death-1 (PD-1) holds the composition for next-generation immunotherapy against tumor [4, 5].

Although CD137 signaling sheds new light on cancer therapy, the potential risk of activating CD137 should not be neglected. For example, it may be an undesirable factor in atherosclerosis (AS). Olofsson et al. found that CD137 is elevated in human plaques and may play an important role in regulating the function of atherosclerosis related cells such as ECs and VSMCs [6]. In animal studies, CD137^−/−^ mice exhibit an obvious reduction of atherosclerosis lesion than WT mice, suggesting that CD137 signal activation may promote plaques formation and destabilization [7–9].

Inflammation is a hallmark of atherosclerosis involving multiple factors and cell types [10]. VSMCs participate in the middle and final phase of plaque formation. VSMCs are highly differentiated cells that are primarily responsible for contraction and the regulation of blood vessel [11]. In middle phase of AS progression, the VSMCs in the media can be transformed from a differentiated phenotype into a dedifferentiated state, which is characterized by accelerated proliferation, migration, and the production of cytokines, to initiate plaque formation once migrated to intima [12]. In final phase, the proliferated VSMCs undergo apoptosis and calcification, which contributes to plaque instability [13].

Recent study suggests that CD137 signaling play an important role in VSMCs function regulation [14]. Normal VSMCs express low CD137 compared with other cells which makes the signaling investigation difficult. To increase the CD137 level, Olofsson and Jung use inflammatory cytokines mix (CM: IL-1*β*, IFN-*γ*, and TNF-*α*) to treat VSMCs. However, we found that the effect of this method depends on the cytokine concentration strictly, and improper concentration may even interfere with the CD137 function. Thus, in this article, we pretreated VSMCs with different concentrations of CM in order to find the reasonable experiment condition for further CD137 study. The better understanding of CD137 signaling on VSMC may help to explore the atherogenic mechanism in AS progression and guide the clinic therapy of CD137 antibody in tumors to avoid the side effect in future.

## 2. Materials and Methods

### 2.1. Mouse Primary Cell Culture

Thirty C57BL/6J mice aged 8 weeks were purchased from Jiangsu University. All the animals were housed under a 12-hour light-dark cycle, and 23 ± 2°C under 55 ± 10% humidity, in normal cages with free access to water and food. The Animal Care and Use Committee of Jiangsu University approved animal experiments. Mouse primary aorta smooth muscle cell was extracted as described previously [14]. Generally speaking, mice were euthanized by CO_2_ and chest was removed. The thoracic aorta was exposed; separate the lipid and fiber membrane from vessel under a surgical microscope. The artery was removed and washed with PBS several times before being treated with type II collagenase. Then, the vessels were cut into pieces and cultivated in cell culture flasks until the tissue block was removed and the 5th–8th generation was used in experiments. Mouse primary cell was cultured in DMED/F12 (Hyclone) medium containing 15% FBS at 37°C and 5% CO_2_.

### 2.2. Cell Treatment

Inflammatory cytokines IL-1*β*, IFN-*γ*, and TNF-*α* are bought from Peprotech. Cells were divided into groups with different concentration gradients (0, 10, 25, 50, 75, and 100 ng/ml) or time points (0, 6, 12, 24, 36, and 48 h), and cell supernatants are collected for sCD137 detection. In CD137 signal activation assay, cells are pretreated with relative CM for 36 h and further treated with or without 10 ng/ml agonist CD137 antibody (R&D) to activate CD137 axis.

### 2.3. Quantitative Real-Time PCR

Total RNA was extracted from VSMCs with TRIzol (Invitrogen). Reverse transcription was carried out with 1 ug RNA using Thermo Fisher RT reagents. CD137 primers are forward: CCTCCAAGTACCTTCTCCAGCA and reverse: CCTCCAAGTACCTTCTCCAGCA. GAPDH primers are forward: GGCATTGCTCTCAATGACAA and reverse: TGTGAGGGAGATGCTCAGTG and were synthesized by Sangon (Shanghai, China).

### 2.4. Western Blot Analysis

The membrane protein and cytoplasm protein were isolated from VSMCs through membrane and cytosol protein extraction kit (Beyotime, China). The proteins were quantified using BCA kit from Vazyme (China), mixed with 5x SDS loading buffer and electrophoresed on a 10% SDS-PAGE gel. The anti-CD137 polyclone antibody (Abcam, USA) was used to detect the monomer or polymer of CD137. Antibodies such as SM-MHC (Abcam, USA), NFATc1 (CST, USA), vimentin (Immunoway, USA), calponin (Abcam, USA), and *α*-SMA (Sigma, USA) were used to observe the phenotype transformation of CD137 axis.

### 2.5. Enzyme-Linked Immunosorbent Assay

Cell culture supernatants were collected from VSMCs treated with gradient cytokines mix or CM + agonist-CD137 antibody. Anti-CD137 ELISA kit (Raybio, USA) was used to detect the sCD137 level secreted from VSMCs under the stimulation CM. IL-2 and IL-6 were purchased from Multi-Science (China) to measure the inflammation response of VSMCs induced by CD137 signal activation. The sensitivity of ELISA kit is 6 pg/ml–1500 pg/ml.

### 2.6. Flow Cytometry

The flow cytometry was performed to observe the CD137 level of membrane, which further validates the results of western blot. Cells were digested and washed with PBS. Anti-CD137-PE (eBioscience, USA) was diluted according to the protocol and was added to 100 *μ*l cell suspension for 30 min at 4°C. After the incubation, the cells were washed one time and then analyzed by flow cytometry (BD Canto).

### 2.7. Transwell and CCK-8 Assay

The cells were pretreated with different concentrations of CM (0, 10, 25, and 50 ng/ml) for 36 h; 5 × 10^4^ cells/ml were plated in 200 *μ*l of DMEM without FBS in the upper chamber, and the lower chamber was filled with 500 *μ*l DMEM containing 2% FBS with/without agonist CD137. The cells were allowed to migrate for 24 h at 37°C in a 5% CO_2_ atmosphere. The cells that remained on the bottom of the lower chamber were fixed with 4% paraformaldehyde, stained with 0.1% crystal violet, and enumerated using Image-Pro Plus 6.0 software.

VSMCs were treated as described previously, 2000 cells were plated in 96-well plate and proliferation was determined using a CCK-8 kit (Vazyme Biotech).

### 2.8. Statistical Analysis

Data are expressed as the mean ± SD of at least three independent experiments (in vitro) and were compared by *t*-test or ANOVA using SPSS version 12.0 (SPSS, Chicago, IL, USA). A two-tailed *P* < 0.05 was considered significant.

## 3. Results

### 3.1. Comparison of CD137 Expression on VSMCs Induced by CM Gradient Treatment

**Figure 1:**
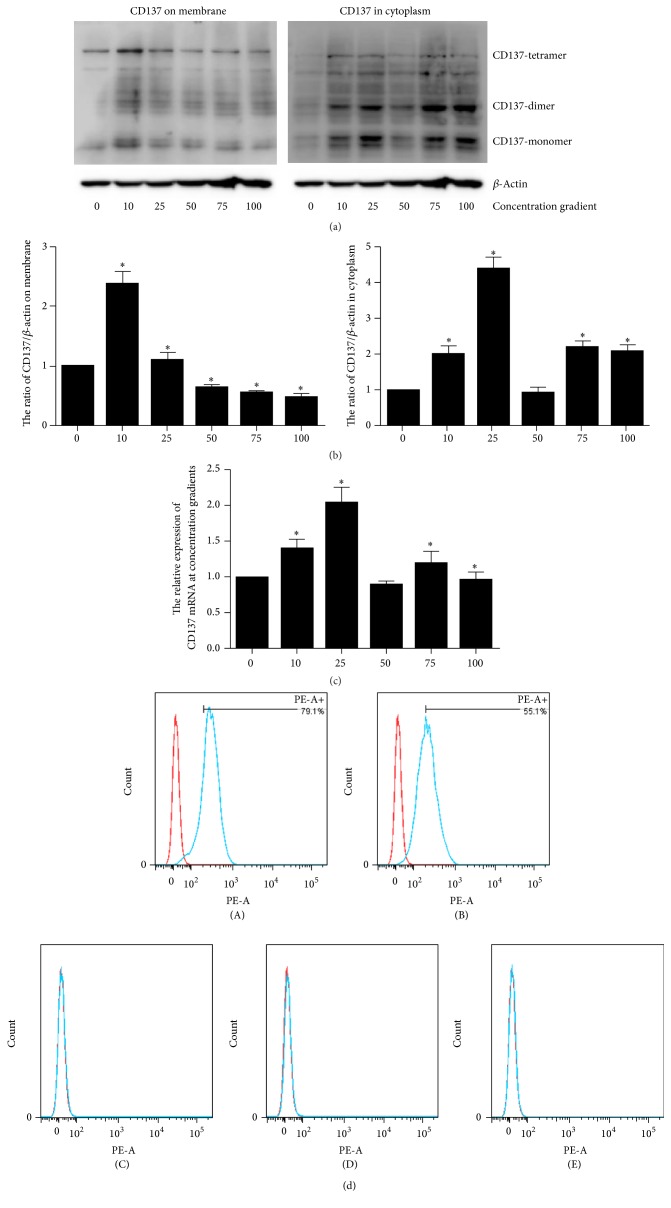
The expression of CD137 treated by CM gradient. (a) The protein level of CD137 on cell surface or in cytoplasm. The different polymer of CD137 exists according to the location. (b) The Gray scale analysis of western blot classified with CD137 location, the mean ± SEM. ^*∗*^*P* < 0.05. (c) The mRNA level of each group. (d) The flow cytometry of CD137 on cell surface compared to 0 ng/ml CM group ((A–E) represents 10, 25, 50, 75, and 100 ng/ml CM groups, resp.).

VSMCs were treated with different concentrations of CM (0, 10, 25, 50, 75, and 100 ng/ml). The mRNA and protein levels of CD137 on membrane or cytoplasm were measured separately (Figure 1). Our data shows that CD137 exists in different forms according to the location; it is more likely to exist as tetramer on membrane but monomer or dimer in cytoplasm. The expression of CD137 elevates under all concentrations of CM stimulation except 50 ng/ml and reaches the peak at 25 ng/ml treatment, but only 10 ng/ml CM can induce the expression of CD137 on membrane. Flow cytometry analysis shows the same results (Figure 1(d)).

### 3.2. Comparison of CD137 Expression on VSMCs at Different Time Points

**Figure 2:**
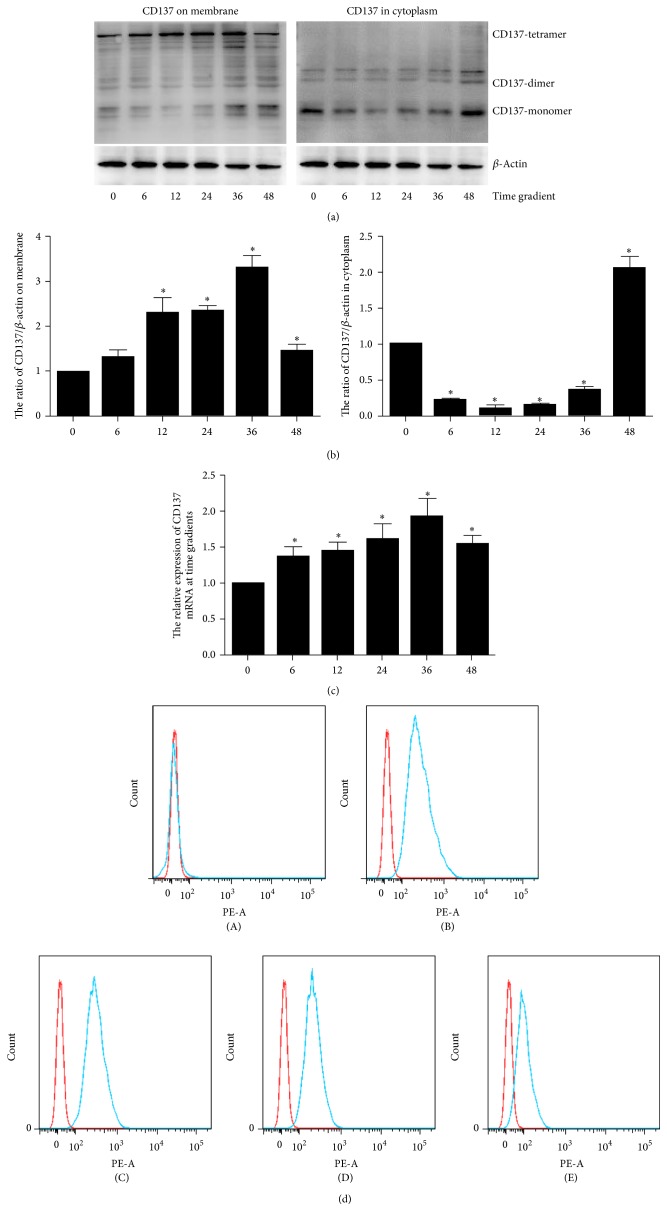
The expression of CD137 in time gradient. (a) The protein level of CD137 on cell surface or in cytoplasm at the time points 0, 6, 12, 24, 36, and 48 h. (b) The Gray scale analysis of according results, the mean ± SEM. ^*∗*^*P* < 0.05. (c) The mRNA level of each group. (d) The flow cytometry of CD137 on cell surface compared to 0 h group ((A–E) represents 6, 12, 24, 36, and 48 h groups).

We choose the 10 ng/ml concentration of CM and detect the expression of CD137 at 0, 6, 12, 24, 36, and 48 h through western blot, Q-PCR, and flow cytometry. The total level of CD137 elevates with time and reaches the peak at 36 h (Figure 2). The flow cytometry and western blot suggest that the membrane CD137 may exist at a high level from 12 h to 36 h but decrease at 48 h. Interestingly, the level of CD137 in cytoplasm is higher in 0 h and 48 h, showing an opposite tendency with membrane CD137.

### 3.3. Detection of sCD137 in Cell Supernatants

**Figure 3:**
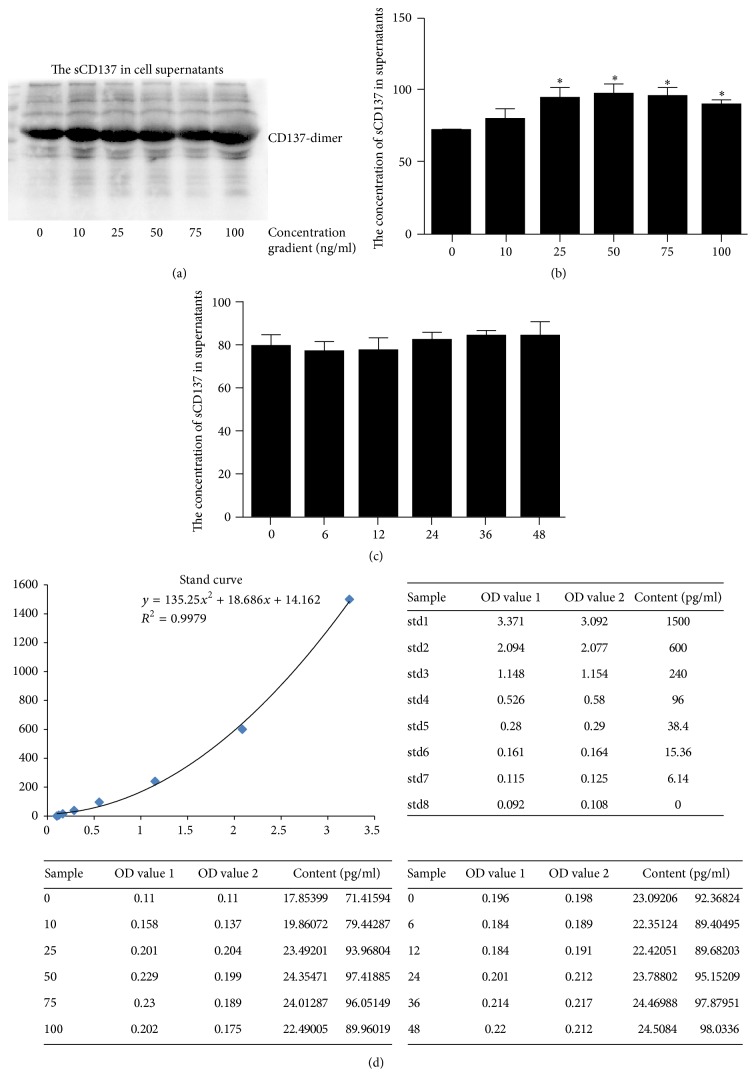
The expression and format of sCD137 in cell supernatant treated with CM. (a) sCD137 exists as dimer which is similar with CD137 in cytoplasm. (b, c) The quantity of sCD137 induced by CM treatment at different concentrations and time points using ELISA, ^*∗*^*P* < 0.05. (d) The stand curve and OD values above.

The CD137 in cytoplasm may be associated with the level of soluble CD137 (sCD137) which is reported to be the potent competitive inhibitor of membrane CD137 [15, 16]. Thus, we use western and ELISA to observe the forms and content of sCD137 secreted from VSMCs induced by CM. Western blot shows that sCD137 in supernatant is dimer, similar to cytoplasm CD137 (Figure 3(a)). ELISA results suggest that the level of sCD137 increases with the CM concentration gradient but shows no difference at different time points (Figures 3(b), 3(c), and 3(d)). The sCD137 levels secreted by VSMC are obviously increased at 25, 50, and 75 ng/ml CM compared to other groups.

### 3.4. VSMCs Phenotype Transformation Induced by CD137 Signaling with Different CM Concentrations

In previous study, our groups found that CD137 signaling can induce phenotype transformation in VSMCs which contributed to atherosclerosis plaque formation. In the present study, we investigated the phenotype in VSMCs to identify the CD137 signaling activation.

**Figure 4:**
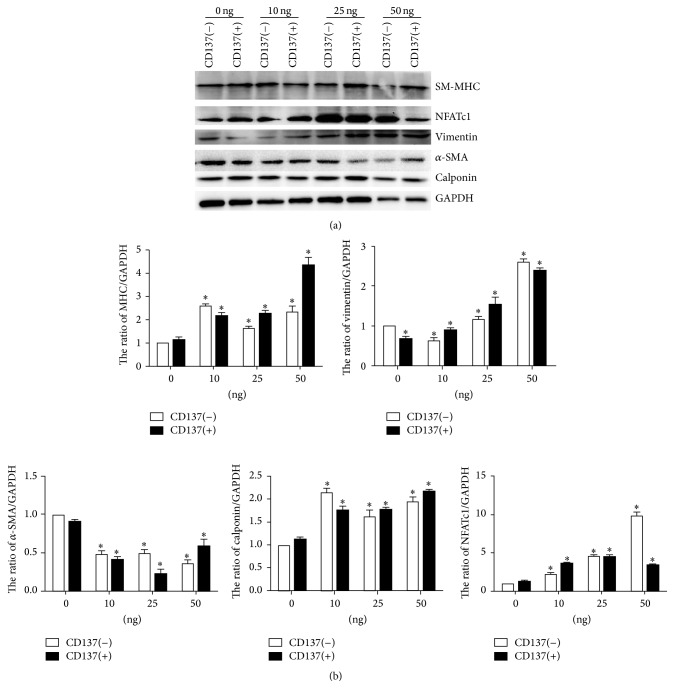
The comparison of phenotype transformation in VSMCs induced by CD137 signaling on different concentration treatment. (a) The western blot of phenotype markers in VSMCs. (b) The Gray scale analysis of western blot, the mean ± SEM, ^*∗*^*P* < 0.05.

Four concentrations (0, 10, 25, and 50 ng/ml) of CM were chosen as pretreatment for 36 h before adding agonist-CD137 antibody to activate CD137 signaling. Western blot shows that VSMCs pretreated with 10 ng/ml CM undergo significantly phenotype transformation induced by agonist-CD137 antibody. Both 10 and 25 ng/ml CM group exhibited VSMCs phenotype transformation; the expression of phenotype proteins such as SM-MHC, *α*-SMA, and calponin decreases while expression of vimentin increases in VSMCs (Figure 4). In 0 ng/ml CM concentration groups, the according proteins show no significant difference. Interestingly, in 50 ng/ml CM group, we observed the opposite effect on VSMCs phenotype. SM-MHC increases while vimentin and the phenotype regulation protein NFATc1 decrease in 50 ng/ml CM group compared to 10 ng/ml group.

### 3.5. VSMCs Migration and Proliferation Induced by CD137 Signaling with Different CM Concentrations

**Figure 5:**
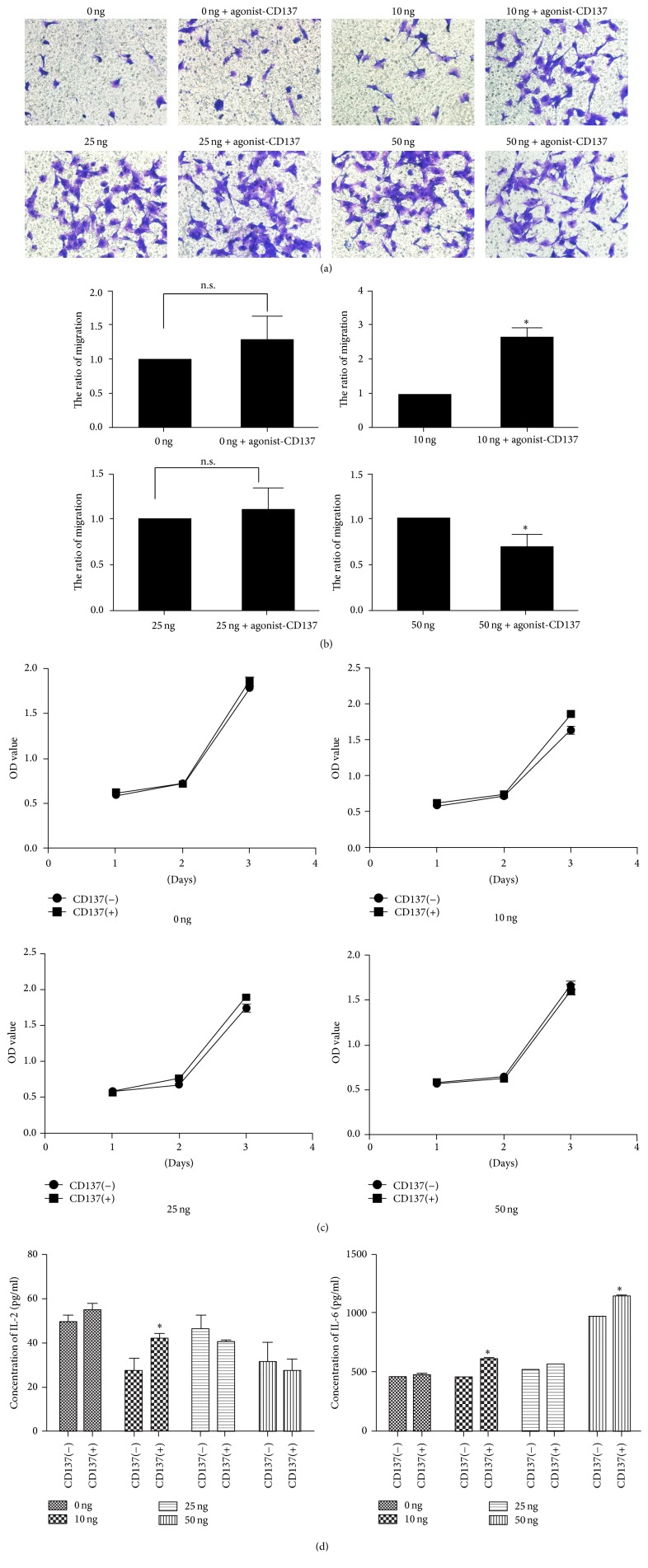
The comparison of cell function changes in VSMCs induced by CD137 signaling on different concentration treatment. (a) The transwell assay of VSMCs with CD137 signaling activation at different CM concentration. (b) The analysis of relative migration ratio in transwell assay, the mean ± SEM. ^*∗*^*P* < 0.05. (c) The cell viability of VSMCs measured by CCK-8 assay during 3 days. (d) The secretion ability of inflammatory cytokines IL-2 and IL-6 induced by CD137 signaling.

The effect of CD137 signaling under different concentrations of CM before treatment was measured by transwell assay to determine the migration ability of cells (Figures 5(a) and 5(b)). In Figure 5, the migration cells number shows no significant difference in 0 ng and 25 ng/ml CM groups when treated with or without agonist-CD137. In 10 ng/ml CM group, CD137 signaling activation induced cell migration while inhibiting it in 50 ng/ml CM group. It should be noted that high concentration (25 ng/ml or 50 ng/ml) CM stimulation can increase basic cell migration.

In cell viability assay, VSMCs in 10 ng/ml CM and 25 ng/ml CM group showed increased proliferation when treated with agonist-CD137 (Figure 5(c)).

### 3.6. VSMCs Secrete IL-2 and IL-6 When CD137 Signaling Is Activated by Different Concentrations of CM

The concentration of IL-2 and IL-6 in supernatant secreted by VSMCs is measured by ELISA (Figure 5(d)). IL-2 and IL-6 levels were significantly elevated when CD137 signaling was activated by 10 ng/ml CM treatment. We also observed a high basic level of IL-6 when treated by 50 ng/ml CM.

## 4. Discussion

CD137 is one of the costimulators of T cells and through binding with CD137 ligand it activates CD137 signaling and promotes proliferation and cytokine production [17]. In recent years, CD137 signaling is regarded as an important target in tumor therapy because it stimulates immune system. For example, combination of agonist-CD137 antibody and PD-1 blockade eradicated tumors through inducing strong immunity in T cells [4]. Moreover, constructed chimeric antigen receptor modified T cells (CART) with anti-CD20 scFv, and human CD137 and CD3*ζ* signaling was proved to be an effective treatment modality in patients with relapsed or refractory aggressive diffuse large B cell lymphomas (DLBCL) [18].

CD137 signaling can enhance immune and inflammation response. But the blessing in tumor therapy may be detrimental in atherosclerosis, a disease which is caused by inflammation and immune overactivation. Many researches, including ours, suggest that activation of CD137 signaling may be a risk in plaque formation. Meanwhile, the study of CD137 in atherosclerosis is still limited due to the low basic level of CD137 expression on several atherosclerosis related cells especially VSMCs [6, 8]. Researchers always pretreated VSMCs with cytokine mix to elevate CD137 expression before studying CD137 signaling, but this method is undefined and without verification. Thus, in this article, we performed a systematic study to validate the effect of the method.

In our study, we found that the concentration of CM is important, because the elevation of CD137 is not linear with CM concentration. In Figure 1, we demonstrate that although 25 ng/ml CM induces the most expression of total CD137, the membrane CD137 that may be functional is elevated upon 10 ng/ml CM stimulation. It should be noticed that CD137 might exist as different forms according to the location and function. For example, the CD137 on membrane is tetramer, the CD137 in cytoplasm is dimer or monomer, and sCD137 always exists as dimer in VSMCs (Figures 1 and 3). Whether CD137 in other tissues is tetramer or dimer and whether different forms of CD137 have different function are not known. It is implied that the different structure of CD137 can be used as a target, which may have the cell specificity to avoid the side effect of agonist-CD137 antibody in clinic trial.

We also detected the time gradient to find the time point that is better for CD137 signaling activation. Interestingly, the expression of membrane CD137 increased from 12 h and reached the top at 36 h while the cytoplasm CD137 had an opposite tendency. The reason for this phenomenon may be that CD137 in cytoplasm undergoes a dynamic translocation to membrane or is secreted to extracellular as sCD137.

As stated above, CM stimulation induced the total CD137 elevation. More than membrane CD137, sCD137 which is regarded as a potent competitive inhibitor of CD137 may also be elevated [15, 16]. Thus, we measure the sCD137 in supernatant by ELISA. Consistent with our hypothesis, sCD137 also increased with the CM gradient. Compared to other concentrations, 10 ng/ml CM shows less sCD137 secretion, which means less interference by CD137 signaling activation.

We finally detect the CD137 signaling activation using agonist-CD137 antibody at different concentrations of CM. Because there has not been an acknowledged standard to measure the CD137 signaling activation in VSMCs [19, 20], we took the VSMCs phenotype transformation and cell function as the indicators which are associated with inflammatory cytokines (IL-2, IL-6) secretion as proved in our previous study. Consistent with our hypothesis, the three indicators exhibit obvious changes induced by agonist-CD137 only at the 10 ng/ml CM pretreatment. Jung et al. used 25 ng/ml CM to pretreated VSMCs for the further CD137 activation and showed a good performance [8]. However, in our study, we found that although 25 ng/ml CM has some effects, further CD137 activation is not stable compared to 10 ng/ml CM pretreatment. The reason for these discrepancies may be due to the actual concentration of CM or cell status. In addition, we found that the response of VSMCs induced by CD137 signaling might be dependent on the CM concentration. For example, VSMCs treated by high concentration of CM (25 ng/ml, 50 ng/ml) may execute even the opposite effect when CD137 signaling is activated. In Figure 4, we showed an opposite tendency in phenotype transformation markers when cells were pretreated by 50 ng/ml CM. The similar phenomenon also exists in cell migration and IL-2 secretion.

Thus, our data suggest that the effect of CM pretreated method before CD137 activation assay depends strictly on the cytokine concentration, and improper concentration may even interfere with the CD137 function. It is worth mentioning that Olofsson and Jung found that CD137 signaling activation induced VSMC apoptosis in VSMCs, but we observed a mild increase in cell viability through CCK-8 assay. The difference may also be attributed to the instability of CM pretreatment.

In this article, we explored the function of different polymers of CD137 and performed an investigation on further validation and improvement of the CM treatment method for CD137 signaling study on VSMCs. We found that 10 ng/ml CM pretreatment is more reasonable than other concentrations when exploring CD137 function in VSMCs. The results may help us to choose a better condition when studying the mechanism of CD137 signaling in AS. More than this, the relationship between inflammatory cytokines and CD137 activation could be a consideration in future clinic CD137 antibody in tumor therapy to avoid side effects such as AS or other inflammation related diseases.
